# The Relationship between PBLS and Osteosarcoma Distribution in Different Subgroups and the Survival and Prognosis of Osteosarcoma

**DOI:** 10.1155/2023/3893134

**Published:** 2023-04-07

**Authors:** Peng Luo, Peng Zhou

**Affiliations:** ^1^Department of Orthopedics, Hubei Provincial Hospital of Integrated Chinese and Western Medicine, Wuhan 430015, Hubei, China; ^2^Department of Orthopedics and Joints, The Third Affiliated Hospital of Soochow University, Changzhou 213000, Jiangsu, China

## Abstract

**Objective:**

To analyze the differences in the distribution of lymphocytes (PBLS) in different subgroups of osteosarcoma (OS) and the predictive value of related parameters on the survival prognosis of OS.

**Methods:**

For retrospective analysis, 80 patients with malignant OS diagnosed and treated in our hospital from June 2016 to June 2017 were selected as the observation group, and 80 patients with benign bone tumors during the same period were selected as the control group. Patients in the observation group were followed up for three years and grouped according to the tumor diameter, stage, metastasis, and prognosis. Fasting venous blood was collected from each group and the levels of CD3+, CD3+CD4+, and CD3+CD8+ were detected. Meanwhile, the ratio of CD4+/CD8+, CD4+/CD3+, and CD8+/CD3+ was calculated and compared. Kaplan–Meier survival curve was used to analyze the relationship between PBLS parameters and OS survival. The area under the curve (AUC), sensitivity, and specificity of each entry index were analyzed by the receiver operating characteristic curve (ROC curve).

**Results:**

The CD3+CD8+ level and CD4+/CD3+ ratio in the observation group were significantly higher than those in the control group (*P* < 0.05). The level of CD3+CD8+ in the patients with tumor diameter ≥ 11 cm was observably higher than that in the patients with tumor diameter <11 cm (*P* < 0.05). The levels of CD3+CD4+ and the ratio of CD4+/CD8 and CD4+/CD3+ of patients in stage III were markedly lower than those of patients in stage II, while the ratio of CD8+/CD3+ and the levels of CD3+CD8+ were prominently higher than those of patients in stage II (*P* < 0.05). The CD3+CD4+ level and CD4+/CD3+ ratio of patients in the metastatic group before treatment, the metastatic group after treatment, and the nonmetastatic group after treatment increased successively, while the ratio of CD4+/CD8+ and CD8+/CD3+ and the level of CD3+CD8+ decreased successively (*P* < 0.05). The CD3+CD4+ level and CD4+/CD3+ ratio in the poor prognosis group were significantly higher than those in the good prognosis group, whereas the ratio of CD8+/CD3+ and CD4+/CD8+ and the level of CD3+CD8+ were significantly lower than those in the poor prognosis group (*P* < 0.05). ROC curve analysis showed that the AUC of CD4+/CD8+ and CD4+/CD3+ in predicting poor prognosis in patients with OS was notably higher than other indicators, which were 0.818 and 0.866, respectively (*P* < 0.05). Kaplan–Meier survival curve results revealed that patients with CD3+CD4+ ≤ 5.15, CD3+CD8+ > 3.85, CD4+/CD8+ ≤ 1.42, CD4+/CD3+ ≤ 0.50, and CD8+/CD3+ > 0.38 had longer survival.

**Conclusion:**

The distribution of PBLS parameters varied widely among different subgroups of OS. Patients with poor prognosis had a higher ratio of CD4+/CD8+ and CD4+/CD3+, which were related to the survival of patients with OS. Moreover, both the ratio of CD4+/CD8+ and CD4+/CD3+ had certain predictive values in terms of survival and prognosis of OS. Therefore, regular clinical monitoring of patients' immune function could help predict disease changes and assess prognosis.

## 1. Introduction

Osteosarcoma (OS) is one of the most common bone malignancies in the clinic, which develops from mesenchymal cell lines. It is characterized by malignant spindle stromal cells that can produce bone-like tissue, which directly or indirectly forms osteoid tissue or bone tissue in the cartilage stage, thereby promoting the rapid growth of tumors. The incidence of OS accounts for about 11.70% of primary bone tumors with the characteristics of high incidence, high metastasis, and poor prognosis. It is more common in children and young adults, thus seriously threatening people's normal growth and development and daily life [1, 2]. With the rapid development of medical technology in recent years, the 5-year survival rate of OS has increased to about 65%, but the metastasis rate after treatment is still as high as 40%. Furthermore, the 5-year survival rate of patients with metastasis is only 25% [3, 4]. Therefore, evaluating the prognostic indicators of patients with OS has become the focus of current medical scholars.

Previous studies have shown that the occurrence and development of tumors are closely related to immune damage and disorders of the body [5, 6]. Meanwhile, the proportion of peripheral blood lymphocytes (PBLS) infiltrated in the tumor microenvironment (TME) of patients with OS is significantly imbalanced, suggesting that PBLS may be associated with the prognosis of OS [7]. PBLS are mainly lymphocytes in the blood circulation, which are composed of T cells, B cells, and NK cells. The PBLS count reflects the changes of some lymphocyte subsets and has been widely used in malignant solid tumors, which is a comprehensive, simple, and dynamic immune evaluation index. Moreover, it has been demonstrated that PBLS mediate the apoptosis of liver cancer cells, and the ratio of PBLS to neutrophils or monocytes is of great value in evaluating the prognosis of liver cancer patients [8]. However, there are only a few studies on PBLS for evaluating the prognosis of OS.

Thus, in this study, 80 patients with OS treated in our hospital from June 2016 to June 2017 were selected as the objects to analyze the difference in PBLS distribution in different subgroups of OS and the predictive value of related parameters on the survival and prognosis of OS, thereby providing a new clinical reference index for the prognosis evaluation of patients with OS.

## 2. Materials and Methods

### 2.1. General Materials

**Figure 1:**
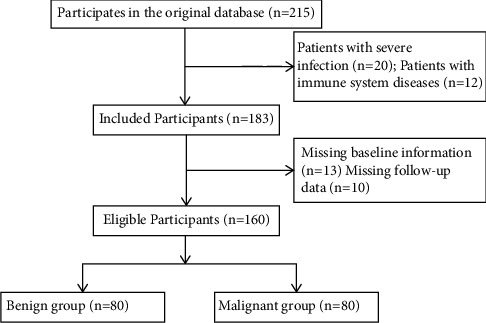
Process of general data selection.

80 patients with OS admitted to our hospital from June 2016 to June 2017 were selected as the observation group and 80 patients with benign bone tumors were selected as the control group during the same period. Inclusion criteria [9]were as follows: All patients were diagnosed as OS by pathological examination; the patients had complete clinical data and were well informed with good compliance to cooperate with the examination and follow-up; the patients signed the informed consent form. Exclusion criteria were as follows: The patients had OS combined with other malignant tumors; the patients were complicated with infection and inflammatory diseases; the patients used antibiotics, immune agents, hormones, and other drugs two weeks before treatment; and the patients were during lactation or pregnancy period. There were 80 cases in the control group, including 45 males and 35 females, with an average age of (23.16 ± 15.85) years. There were 80 cases in the observation group, including 47 males and 33 females, with an average age of (23.28 ± 12.04) years old. The patients in the observation group were further divided into subgroups according to tumor diameter (diameter <11 cm and diameter ≥ 11 cm), stage (stage II and stage III), and metastasis (metastasis before treatment, metastasis after treatment, and no metastasis after treatment). There was no significant difference in age and gender between the groups (*P* > 0.05) (Table 1). The general data selection is shown in Figure 1. All the experiments were approved by the ethics committee of our hospital.

### 2.2. Outcome Measurements

The PBLS parameter detection [10]: the fasting venous blood of patients in each group before treatment on the day of admission was collected and centrifuged at 3000 r/min for 30 min. The supernatant was carefully collected and stored at −80°C for subsequent assays and avoided repeated freezing and thawing. 20 *μ*L of fluorescent-labeled mouse-anti-human CD4+-FITC/CD8-PE/CD3-PE-Cy5 monoclonal antibodies (IBIO-18326R, IBIO, Jiangxi, China) were gently added into 100 *μ*L whole blood and mixed softly. The tube was left standing in the dark at 18–25°C for 20 min. Then, the red blood cells were dissolved with an erythrocyte hemolytic agent (mlE4333, Mlbio, Shanghai, China) and the tube was centrifuged at 1200 pm for 5 min. The supernatant was discarded, and 500 *μ*L of IPBS was added to resuspend the cells. The levels of CD3+ (%), CD3+CD4+ (%), and CD3+CD8+ (%) in IPBs suspension cells were detected using flow cytometry (130-109-803, Miltenyi Biotec, Germany). Meanwhile, the ratios of CD4+/CD8+, CD4+/CD3+, and CD8+/CD3+ in each group were calculated and compared. At least 1000 cells were detected for each sample. The data were obtained and analyzed by FCM software SYSTEM™ II to obtain the percentage of fluorescent-labeled positive cells. The above flow cytometry detection was conducted in the best state when the light path of flow-check standard fluorescent microspheres was controlled at CV < 2%.

Prognosis follow-up: all patients were followed up during our hospital visit. They were followed up every three months in the first two years and every six months in the next year. The follow-up included outpatient, inpatient, and telephone follow-ups. The survival of all patients was recorded. The endpoint of follow-up was the death of the patient or the end of follow-up. The survival of all patients was recorded, in which the patients who died during the follow-up period were in the poor prognosis group, and the patients who survived during the follow-up period were in the good prognosis group.

### 2.3. Statistical Analysis

SPSS 20.0 software was used to analyze the experimental data. Measurement data such as the age and PBLS parameters were expressed in (‾*x* ± *s*) and were compared using the *t*-test between two groups and compared with the repeated measures analysis of variance among multiple groups. The enumeration data such as sex, tumor diameter, stage, and metastasis were expressed in (%) and were compared using the *χ*^2^ text. The relationship between PBLS parameters and the survival time of OS was analyzed using the Kaplan–Meier survival curve. The area under the curve (AUC), sensitivity, and specificity of each entry index were analyzed using a receiver operating characteristic curve (ROC curve). *P* < 0.05 indicated that the statistical results were statistically significant.

## 3. Results

### 3.1. Comparison of PBLS Parameters between Two Groups

The CD3+CD8+ level and CD4+/CD3+ ratio of patients in the observation group were significantly higher than those of patients in the control group (*P* < 0.05). There was no significant difference in the levels of CD3+, CD3+CD4+, and CD3+CD8+ and the ratio of CD8+/CD3+ between the two groups (*P* > 0.05) (Table 2).

### 3.2. Comparison of PBLS Parameters in Different Subgroups of OS Patients

The level of CD3+CD8+ of patients in the diameter ≥ 11 cm group was significantly higher than that in the diameter <11 cm group (*P* < 0.05). The level of CD3+CD4+ and the ratio of CD4+/CD8+ and CD4+/CD3+ of patients in the stage III group were prominently lower than those of patients in the stage II group, while the ratio of CD8+/CD3+ and the level of CD3+CD8+ were notably higher than those of patients in the stage II group (*P* < 0.05). The CD3+CD4+ level and CD4+/CD3+ ratio of patients in the metastatic group before treatment, the metastatic group after treatment, and the nonmetastatic group after treatment increased successively, while the ratio of CD4+/CD8+ and CD8+/CD3+ and the level of CD3+CD8+ decreased successively (*P* < 0.05) (Tables 3–5).

### 3.3. Comparison of PBLS Parameters in Patients with Different Prognosis

During the follow-up period of this experiment, 18 patients died, and the mortality rate was 22.50%. The CD3+CD4+ level and CD4+/CD3+ ratio of patients in the poor prognosis group were significantly higher than those of patients in the good prognosis group, whereas the ratio of CD8+/CD3+ and CD4+/CD8+ and the level of CD3+CD8+ of patients in the poor prognosis group were observably lower than those of patients in the good prognosis group (*P* < 0.05) (Table 6).

### 3.4. Analysis of the Value of PBLS Parameters in Predicting Survival

**Figure 2:**
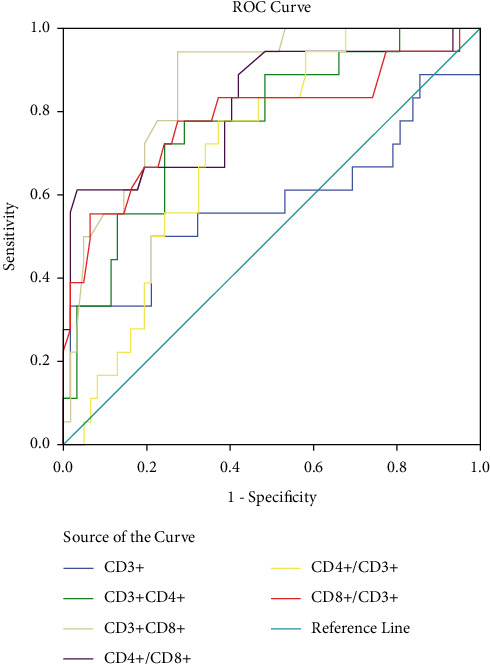
ROC curve analysis.

ROC curve analysis showed that the AUC of CD4+/CD8+ and CD4+/CD3+ in predicting poor prognosis in patients with OS was much higher than other indicators, with the AUC of 0.818 and 0.866, respectively, the sensitivity of 80.12% and 90.52%, respectively, the specificity of 85.16% and 86.35%, respectively, and the threshold of 1.32 and 0.56, respectively (*P* < 0.05) (Table 7 and Figure 2).

### 3.5. Correlation between PBLS Parameters and Survival Time of OS

**Figure 3:**
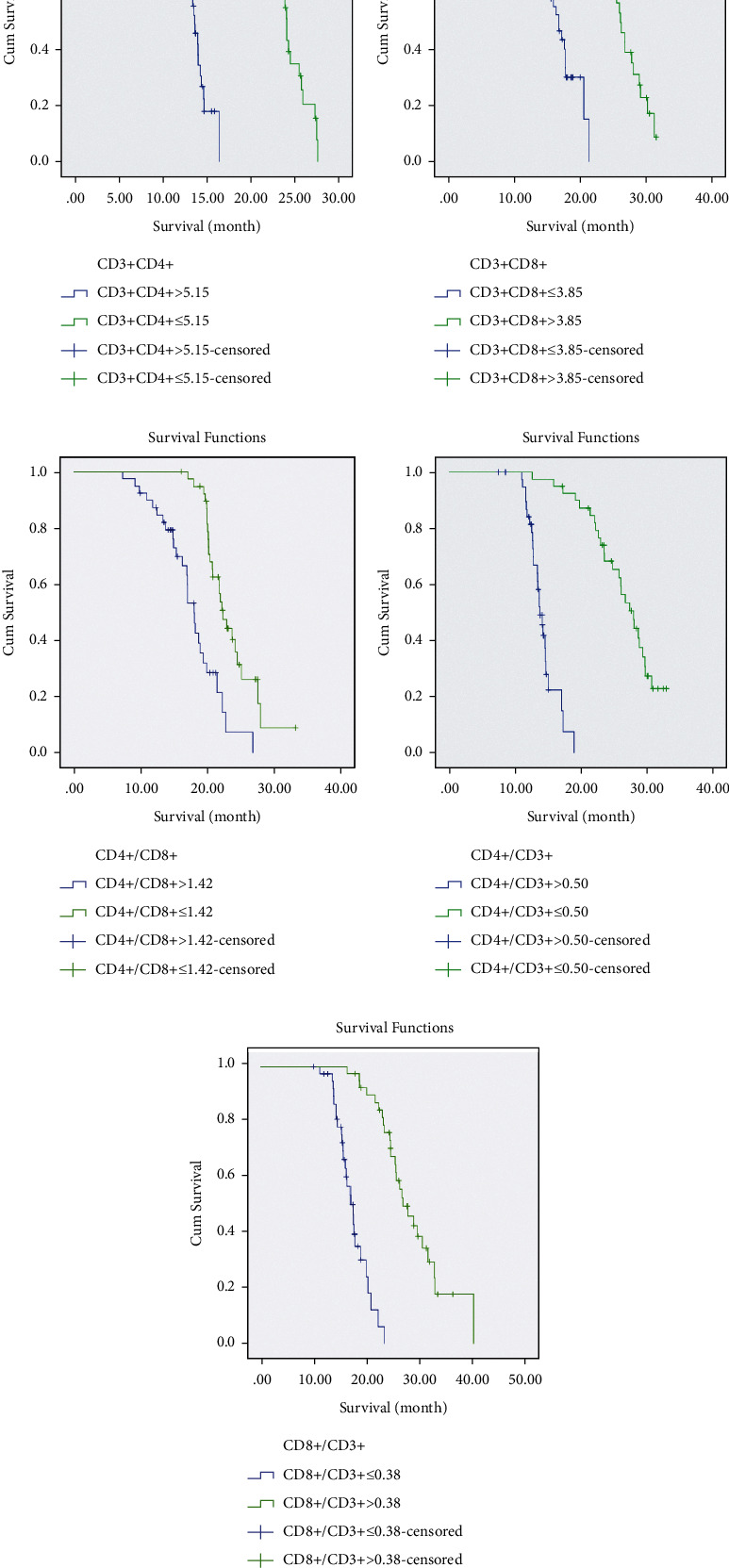
Kaplan–Meier survival curve analysis of the relationship between PBLS parameters and the survival time of OS. (a) Survival curve of different CD3+CD4+ levels. (b) Survival curve of different CD3+CD8+levels. (c) Survival curve of different ratios of CD4+/CD8+. (d) Survival curve of different ratios of CD4+/CD3+. (e) Survival curve of different ratios of CD8+/CD3+.

80 patients with OS were followed up for 36 months, with a median survival time of 18.25 months. The results of the Kaplan–Meier survival curve grouped according to different variables revealed that the survival time of patients with CD3+CD4+ ≤ 5.15 was significantly higher than that of patients with CD3+CD4+  5.15, the survival time of patients with CD3+CD8+  3.85 was prominently higher than that of patients with CD3+CD8+ ≤ 3.85, the survival time of patients with CD4+/CD8+ ≤ 1.42 was notably higher than that of patients with CD4+/CD8+  1.42, the survival time of patients with CD4+/CD3+ ≤ 0.50 was observably higher than that of patients with CD4+/CD3+  0.50, and the survival time of patients with CD8+/CD3+  0.38 was markedly higher than that of patients with CD8+/CD3+ ≤ 0.38 (all *P* < 0.05) (Table 8 and Figure 3).

## 4. Discussion

OS is one of the most common primary bone malignancies worldwide, mostly in adolescents and the elderly. Previous studies have shown that the occurrence of OS is closely related to hormonal changes in puberty and physiological bone growth. The early symptoms are mostly pain and swelling at the lesion site. Due to the atypical symptoms, patients cannot be concerned, and most patients have early metastases when they visit a doctor, so the treatment of OS is difficult [8]. Immunotherapy has historically been one of the most widely used strategies to treat many types of cancers, and therapies associated with T cell responses, such as immune checkpoint inhibitors and chimeric antigen receptor T cell therapy, have been considered good options for certain cancers. Although neoadjuvant chemotherapy for OS has shown promising results, overall survival in patients with metastases has remained low over the past 30 years. At present, there is still a lack of unified and effective disease prognostic indicators, especially immune-related prognostic indicators, which are of great significance for improving the prognosis of patients and prolonging the lives of patients.

It has been revealed that OS can induce different degrees of immune disorders in the body [11]. PBLS includes available T cells, B cells, and NK cells, which play an important role in mediating cellular immunity, especially in antitumor immunity. In cancer patients with low immune function, tumor cells are easy to escape the immune monitoring of the body, thus creating conditions for the development of tumors. CD3 represents all T lymphocytes, which can also be divided into CD4+ T cells and CD8+ T cells. Among them, CD4+T cells mediate the functions of helper T cells (Th), NKT cells, macrophages, and dendritic cells, while CD8+ lymphocytes mediate the functional changes of cytotoxic T lymphocytes (CTL) [12, 13]. The PBLS detection can evaluate the changes of lymphocyte subsets and the steady-state changes of lymphocyte subsets. Meanwhile, PBLS detection also has the advantages of simple operation, convenient acquisition, low cost, and repeatable detection; thus, it is more suitable as an immune indicator for monitoring disease changes and prognosis. In the present experiment, the PBLS indexes of OS patients with different tumor diameters, Enneking stages, and metastases were significantly different, indicating that there was a certain degree of immune damage in OS patients. All PBLS indexes reflected the immune function status of T lymphocytes in the body; thus, the abnormal expression of PBLS indexes was closely related to the development of OS.

The change of CD4+/CD8+ reflects the relationship between Th and CTL. Th cells can be divided into different lineage subgroups, and different lineages can be transformed into each other, so it is difficult to detect Th cell changes. However, CTL is the core part of tumor cell immunity, which can kill tumor cells by recognizing tumor antigen peptides. Generally, more tumor cells in the body secreting more tumor antigen peptides and CTL cells will be significantly activated and proliferated [14, 15]. Therefore, the lower the CD4+/CD8+ ratio is, the worse the prognosis of patients is. In the present study, the patients in the poor prognosis group had much higher level of CD3+CD4+ and a ratio of CD4+/CD3+ and a lower ratio of CD8+/CD3+ and CD4+/CD8+ and a level of CD3+CD8+ than the patients in the good prognosis group, suggesting that the changes of PLBS could reflect the immune damage of the body and had a certain relationship with the 5-year survival of patients with OS. It has been found that peripheral CD4+CXCR5+ T cells participate in the pathogenesis and progress of OS, and patients with high tumor grade show a significant increase in the percentage of CD4+CXCR5+ T cells compared with patients with low OS grade [16]. OS has the characteristics of high malignancy, high metastasis rate, and poor prognosis, which seriously threaten people's lives, health, and quality of life. Early prediction of the prognosis of OS is helpful to prolong the lives of patients. In order to further verify the relationship between the changes of PLBS and the prognosis of OS, the ROC curve was established. The analysis showed that the AUC of CD4+/CD8+ and CD4+/CD3+ in predicting the poor prognosis of OS patients had a higher AUC than other indicators, which were 0.818 and 0.866, respectively, which further indicated that the changes in CD4+/CD8+ and CD4+/CD3+ levels were closely related to the prognosis of patients with OS. Pratt et al. [17] and Lee et al. [18] also confirm that the peripheral CD4+/CD8+ ratio is a simple, reliable, and economic prognostic indicator for the survival prediction of patients with advanced OS, which can be combined with clinical indicators to establish a better prognostic model. This present study suggested that the change of CD4+/CD8+ ratio of PBLS had a certain predictive value in predicting the prognosis of patients with OS, which could be helpful to guide the treatment plan, prolong the life of patients, and improve the prognosis of patients.

In conclusion, the distribution of PBLS parameters varied widely among different subgroups of OS. Among them, patients with poor prognosis had higher levels of CD4+/CD8+ and CD4+/CD3+, which were related to the survival of patients with OS. Moreover, both the levels of CD4+/CD8+ and CD4+/CD3+ had certain predictive values in terms of survival and prognosis of OS. Therefore, regular clinical monitoring of patient immune function could help predict disease changes and assess prognosis. However, this study still has some limitations. The research objects are all patients in our hospital, not a random sample of the entire target population. Thus, there may be selection bias in the selection of research objects, which affects the research results. In the following study, the experimental objects and research time will be expanded to further explore the potential mechanisms. In addition, the role of PBLS parameters in the invasion and metastasis of OS also requires further research at a later stage.

## Figures and Tables

**Table 1:** Comparison of general data of two groups of patients ($\bar{x}$ ± *s*).

Index		Control group (*n* = 80)	Observation group (*n* = 80)	*t/χ* ^2^	*P*
Age (year)		23.16 ± 15.85	23.28 ± 12.04	0.054	0.957
BMI value (kg/m^2^)		22.63 ± 1.85	22.45 ± 2.33	0.541	0.589
Gender	Male	45 (56.25%)	47 (58.75%)	0.102	0.749
	Female	35 (43.75%)	33 (41.25%)		
Hypertension history		8 (10.00%)	9 (11.25%)	0.066	0.798
History of diabetes		4 (5.00%)	2 (2.50%)	0.693	0.405
Coronary heart disease		5 (6.25%)	3 (3.75%)	0.526	0.468
Smoking history		8 (10.00%)	10 (12.50%)	0.250	0.617
History of drinking		7 (8.75%)	8 (10.00%)	0.074	0.786

**Table 2:** Comparison of PBLS parameters between two groups ($\bar{x}$ ± *s*).

Index	The control group (*n* = 80)	The observation group (*n* = 80)	*t*	*P*
CD3+ (%)	12.05 ± 2.52	12.85 ± 6.25	1.061	0.290
CD3+CD4+ (%)	6.89 ± 2.85	6.65 ± 3.25	0.496	0.620
CD3+CD8+ (%)	4.19 ± 2.63	4.85 ± 2.46	1.639	0.103
CD4+/CD8+	1.62 ± 0.85	1.36 ± 0.25	2.624	0.010
CD4+/CD3+	0.58 ± 0.12	0.52 ± 0.14	2.910	0.004
CD8+/CD3+	0.36 ± 0.05	0.38 ± 0.12	1.376	0.171

**Table 3:** Comparison of PBLS parameters in patients with OS of different diameters ($\bar{x}$ ± *s*).

Index	Group of diameters < 11 cm (*n* = 42)	Group of diameters ≥ 11 cm (*n* = 38)	*t*	*P*
CD3+ (%)	11.82 ± 5.29	13.95 ± 4.85	1.870	0.065
CD3+CD4+ (%)	6.75 ± 2.15	6.48 ± 1.25	0.677	0.500
CD3+CD8+ (%)	4.12 ± 1.85	6.23 ± 2.15	4.717	<0.001
CD4+/CD8+	1.46 ± 0.45	1.28 ± 0.63	1.481	0.142
CD4+/CD3+	0.55 ± 0.12	0.50 ± 0.15	1.653	0.102
CD8+/CD3+	0.35 ± 0.36	0.42 ± 0.25	0.999	0.320

**Table 4:** Comparison of PBLS parameters in patients with OS at different stages ($\bar{x}$ ± *s*).

Index	Stage II group (*n* = 61)	Stage III group (*n* = 19)	*t*	*P*
CD3+ (%)	13.26 ± 2.46	12.09 ± 2.19	1.855	0.067
CD3+CD4+ (%)	6.82 ± 2.15	5.02 ± 2.16	3.183	0.002
CD3+CD8+ (%)	4.06 ± 2.15	5.36 ± 1.85	2.373	0.020
CD4+/CD8+	1.68 ± 0.15	1.02 ± 0.19	15.689	<0.001
CD4+/CD3+	0.58 ± 0.24	0.40 ± 0.19	2.986	0.004
CD8+/CD3+	0.31 ± 0.15	0.49 ± 0.24	3.916	<0.001

**Table 5:** Comparison of PBLS parameters in OS patients with different metastatic conditions ($\bar{x}$ ± *s*).

Index	Pretreatment metastasis group (*n* = 17)	Posttreatment metastasis group (*n* = 26)	Posttreatment nonmetastasis group (*n* = 37)	*F*	*P*
CD3+ (%)	12.36 ± 2.15	12.79 ± 0.14	12.98 ± 2.15	0.720	0.492
CD3+CD4+ (%)	5.06 ± 1.45	6.38 ± 2.15^a^	8.51 ± 2.15^ab^	19.250	<0.001
CD3+CD8+ (%)	5.85 ± 1.26	4.41 ± 1.28^a^	3.29 ± 2.48^ab^	10.440	<0.001
CD4+/CD8+	1.68 ± 0.35	1.41 ± 0.24^a^	1.02 ± 0.21^ab^	43.660	<0.001
CD4+/CD3+	0.30 ± 0.16	0.46 ± 0.25^a^	0.61 ± 0.25^ab^	10.650	<0.001
CD8+/CD3+	0.53 ± 0.12	0.45 ± 0.16^a^	0.35 ± 0.08^ab^	14.350	<0.001

*Note.* ^a^ *P* < 0.05 compared with the pretreatment metastasis group; ^b^*P* < 0.05 compared with the posttreatment metastasis group.

**Table 6:** Comparison of PBLS parameters in patients with different prognosis ($\bar{x}$ ± *s*).

Index	Good prognosis group (*n* = 62)	Poor prognosis group (*n* = 18)	*t*	*P*
CD3+ (%)	12.63 ± 1.05	13.01 ± 2.12	1.048	0.298
CD3+CD4+ (%)	5.86 ± 2.15	8.05 ± 1.63	3.994	<0.001
CD3+CD8+ (%)	5.26 ± 1.85	3.56 ± 2.85	3.011	0.004
CD4+/CD8+	1.21 ± 0.16	1.58 ± 0.28	7.174	<0.001
CD4+/CD3+	0.39 ± 0.25	0.62 ± 0.15	3.704	<0.001
CD8+/CD3+	0.56 ± 0.28	0.34 ± 0.16	3.177	0.002

**Table 7:** Analysis about the value of PBLS parameters in predicting survival.

Index	AUC	95% CI	Threshold	*P*	Sensitivity (%)	Specificity (%)
CD3+	0.529	0.401∼0.762	8.96	0.294	56.32	52.10
CD3+CD4+	0.774	0.652∼0.896	5.12	<0.01	73.52	64.15
CD3+CD8+	0.712	0.595∼0.829	7.81	0.006	63.26	62.10
CD4+/CD8+	0.818	0.693∼0.943	1.32	<0.01	80.12	90.52
CD4+/CD3+	0.866	0.783∼0.950	0.56	<0.01	85.16	86.35
CD8+/CD3+	0.784	0.643∼0.926	0.41	<0.01	80.26	78.69

**Table 8:** Difference in each factor.

Factors	*P*	HR	95% CI
CD3+CD4+ ≤ 5.15 vs. CD3+CD4+ > 5.15	<0.001	4.285	1.236∼5.748
CD3+CD8+ > 3.85 vs. CD3+CD8+ ≤ 3.85	<0.001	3.533	1.362∼4.215
CD4+/CD8+ ≤ 1.42 vs. CD4+/CD8+ > 1.42	<0.001	4.159	1.005∼8.415
CD4+/CD3+ ≤ 0.50 vs. CD4+/CD3+ > 0.50	<0.001	1.753	0.857∼2.002
CD8+/CD3+ > 0.38 vs. CD8+/CD3+ ≤ 0.38	<0.001	3.241	2.065∼4.296

## Data Availability

All data, models, and code generated or used during this study are included within the article.
